# Surgical and Surgeon-Related Factors Related to Long-Term Survival in Esophageal Cancer: A Review

**DOI:** 10.1245/s10434-019-07966-9

**Published:** 2019-11-05

**Authors:** Sheraz R. Markar, Jesper Lagergren

**Affiliations:** 1 Upper Gastrointestinal Surgery, NS 67, Department of Molecular Medicine and Surgery, Karolinska Institutet, Stockholm, Sweden; 2 Department of Surgery and Cancer, Imperial College London, London, UK; 3 School of Cancer and Pharmaceutical Sciences, King’s College London, London, UK

## Abstract

Esophagectomy is the mainstay of curative treatment for most patients with a diagnosis of esophageal cancer. This procedure needs to be optimized to secure the best possible chance of cure for these patients. Research comparing various surgical approaches of esophagectomy generally has failed to identify any major differences in long-term prognosis. Comparisons between minimally invasive and open esophagectomy, transthoracic and transhiatal approaches, radical and moderate lymphadenectomy, and high and moderate hospital volume generally have provided only moderate alterations in long-term survival rates after adjustment for established prognostic factors. In contrast, some direct surgeon-related factors, which remain independent of known prognostic factors, seem to influence the long-term survival more strongly in esophageal cancer. Annual surgeon volume is strongly prognostic, and recent studies have suggested the existence of long surgeon proficiency gain curves for achievement of stable 5-year survival rates and possibly also a prognostic influence of surgeon age and weekday of surgery. The available literature indicates a potentially more critical role of the individual surgeon’s skills than that of variations in surgical approach for optimizing the long-term survival after esophagectomy for esophageal cancer. This finding points to the value of paying more attention to how the skills of the individual esophageal cancer surgeon can best be achieved and maintained. Careful selection and evaluation of the most suitable candidates, appropriate and structured training programs, and regular peer-review assessments of experienced surgeons may be helpful in this respect.

Esophagectomy remains the mainstay treatment for most esophageal cancer patients selected to undergo curatively intended treatment.^1^ Studies aimed at improving the outcomes of esophageal cancer surgery often have focused on short-term postoperative mortality as the main outcome, whereas fewer studies have examined how variations in surgical factors influence long-term survival. Yet, improvements in postoperative mortality rates, with 30-day mortality rates currently lower than 5% even in unselected settings,^2^^,^^3^ indicate that any potential further improvements in short-term mortality would change the absolute number of saved lives only to a limited extent.

The high risk of mortality from tumor recurrence, with 60–70 % of surgically treated patients dying during the 5 years after surgery,^2^^,^^3^_ENREF_2 suggests that many more deaths attributable to esophageal cancer may be avoided if more research is devoted to improving the long-term oncologic outcomes after esophagectomy.^1^

Another concern is that relatively limited research has been devoted to assessing the prognostic role of surgical factors for esophageal cancer patients, whereas much more research has been invested in pharmacologic and other nonsurgical treatments. Although it is well-established that neoadjuvant chemotherapy or chemoradiotherapy can improve survival,^4^ the effect on survival still is limited, and a significant proportion of patients do not benefit from such therapy at all. Surgical factors may play an important prognostic role for many patients.^1^

This review evaluates the existing literature that examines how variations in surgical and surgeon-related factors may influence the long-term survival of patients who undergo surgery for esophageal cancer.

## Observations

### Surgical Approaches in Relation to Long-Term Survival in Esophageal Cancer

Efforts to improve the surgery aimed at offering better long-term survival in esophageal cancer have been devoted mainly to the role of various surgical approaches and techniques. The optimal surgical approach in the treatment of esophageal cancer is a matter of much debate. This review evaluates the existing literature addressing three key questions in relation to long-term survival: (1) Is minimally invasive esophagectomy better than open surgery? (2) Is the transthoracic approach better than the transhiatal approach? (3) Is extensive lymphadenectomy better than moderate lymphadenectomy?

#### *Minimally Invasive Versus Open Esophagectomy*.

Only two European randomized controlled trials have compared long-term survival after minimally invasive and open surgery for esophageal cancer. The Dutch TIME-trial showed the 3-year overall survival in the totally minimally invasive esophagectomy group (59 patients) to be 40.4% versus 50.5% in the open esophagectomy group (56 patients), but this indication of a difference failed to reach statistical significance in the multivariable regression model (hazard ratio [HR], 0.88; 95% confidence interval [CI], 0.54–1.44).^5^ The French MIRO trial indicated a better 3-year overall survival in the hybrid minimally invasive esophagectomy group (103 patients) than in the open esophagectomy group (104 patients) (67.0% vs. 54.8%; HR, 0.67; 95% CI, 0.44–1.00).^6^

A recent meta-analysis including 55 studies with 14,592 patients showed an 18% reduction in 5-year all-cause mortality after minimally invasive versus open esophagectomy.^7^ The randomized controll trials (RCTs) and meta-analysis suggest that a minimally invasive approach to esophagectomy may be associated with an improved long-term survival, but this needs to be examined in further trials with larger samples before any such survival benefit can be established. Additionally, the mechanism of any such survival benefit remains unknown, although it is possible that immunologic factors related to less extensive procedures or a reduced risk of complications and reoperations after minimally invasive approaches may be involved.^8^^–^10

#### *Transthoracic Versus Transhiatal Approach*.

A Dutch RCT comparing transhiatal patients (*n* = 106) with transthoracic esophagectomy patients (*n* = 114) showed no difference in overall or disease-free survival.^11^^,^^12^ Similarly, a systematic review and meta-analysis of 52 studies and 5905 patients showed no difference in 5-year survival between these approaches, although the extent of lymphadenectomy (with only 4 studies reporting a lymph node yield of ≥ 20) and the reported surgical quality (with no study satisfying six minimum surgical quality standards identified) were suboptimal in both groups.^13^ A more recent meta-analysis also confirmed these findings of no significant differences in prognosis between transhiatal and transthoracic esophagctomy for esophagogastric junctional cancers.^14^

#### *Radical Versus Moderate Lymphadenectomy*.

Comparison of radical and moderate lymphadenectomies, a controversial issue for several years, has become increasingly more complex with the introduction of neoadjuvant chemoradiotherapy to improve locoregional control of tumor growth. A problem with interpretation of these findings is the lack of adjustment for surgeon volume, which correlates with the extent of lymphadenectomy.

A population-based Swedish cohort study of 1044 patients showed that after adjustment for known prognostic factors, including surgeon volume, more extensive or radical lymph node clearance during surgery for esophageal cancer failed to improve the 5-year survival (HR, 1.00; 95% CI, 0.99–1.01, multi-variate analysis).^15^ A cohort study of 606 patients from a large single-center cohort in the United Kingdom, similarly showed that the extent of lymphadenectomy during surgery did not influence 5-year all-cause or disease-specific mortality when control was used for surgeon volume.^16^

A recent meta-analysis of 26 studies and 48,612 patients showed that a higher lymph node yield was associated with decreased overall mortality among all esophagectomy patients (HR, 0.81; 95% CI, 0.74–0.87) and in a subset analysis of patients receiving neoadjuvant therapy (HR, 0.82; 95% CI, 0.73–0.92),^17^ but most studies did not adjust for surgeon volume. A further recent Dutch population-based cohort study of 2698 patients identified a survival benefit of lymph node yield specifically after neoadjuvant chemoradiotherapy (HR, 0.84; 95% CI, 0.78–0.90).^18^ Thus, the controversy about the prognostic role of more or less extensive lymphadenectomy persists, although parts of any survival benefits with extensive lymphadenectomy may be explained by surgeon volume.

#### *Summary of Surgical Approaches*.

Comparisons of minimally invasive surgery with open surgery, transthoracic with transhiatal approach, and extensive with moderate lymphadenectomy generally have provided minor alterations in long-term survival after adjustment for prognostic factors. Although research efforts aimed at improving the overall long-term survival after esophageal cancer surgery have focused mainly on comparing various surgical approaches, these comparisons generally have resulted in minor or no differences.

### Surgeon-Related Factors in Relation to Long-Term Survival in Esophageal Cancer

Compared with assessments of surgical approach, the influence of direct surgeon-related factors in relation to survival after esophageal cancer surgery has been studied less often. Four surgeon-related questions may be of special relevance for the chance of long-term survival after esophagectomy: (1) What is the role of annual hospital volume versus surgeon volume? (2) How does the learning period influence the prognosis? (3) Does the age of the surgeon influence the long-term outcomes for patients? (4) Does timing of the surgery during the working week or holiday periods influence the long-term survival after surgery for esophageal cancer?

Hospital Versus Surgeon Volume. Studies have consistently identified the benefits of high hospital and surgeon volumes in reducing short-term mortality after esophageal cancer surgery.^19^,^20^ Regarding long-term survival, a large cohort study (1335 patients) and a systematic review and meta-analysis of 16 studies considered both hospital volume and surgeon volume and showed high correlation between these two factors. Nevertheless, although high surgeon volume showed a clear and independent survival benefit, the prognostic role of hospital volume became weak or disappeared after adjustment for surgeon volume.^21^,^22^

Proficiency Gain Curves. In more recent years, increasing attention has been paid to adverse patient outcomes during the time when a surgeon gains proficiency as an independent surgeon in undertaking a complex cancer surgery, which can be modeled as a proficiency gain curve (“learning curve”). Multi-center and national cohort studies have identified highly variable lengths in the period of proficiency gain as surgeons perform open and minimally invasive esophagectomy for short-term outcomes (Table 1).^23^^,^^24^ A single-center study and a single-surgeon practice showed improvements in patients’ long-term survival during a 7-year period as the surgeon continued to improve his operative performance.^25^ A national population-based cohort study showed that the period of proficiency gain in Sweden for open esophagectomy was 59 cases for a plateau in 5-year all-cause mortality, with substantial improvement in 5-year survival from 19.1 to 31.4 %.^26^ Further investigation has shown this period of proficiency gain to be modifiable. A higher frequency of esophagectomies during a shorter period and surgeons starting at a younger age shorten the proficiency gain period and thus the adverse outcomes experienced by patients during this time.^27^

**Table 1:** Variability in length of the proficiency gain curves observed with esophagectomy for cancer

Publication	Data set origin	Procedure	Outcome studied	Length of proficiency gain curve (cases)
Mackenzie et al^23^	England	Minimally invasive esophgectomy	30-Day mortality	19
			90-Day mortality	17
			Re-intervention	19
			Conversion	6
			Hospital stay	5
van Workum et al^24^	European multi-center	Minimally invasive esophagectomy	Anastomotic leak, Textbook outcome & operative time	119
Markar et al^26^	Sweden	Open esophagectomy	30-Day mortality	15
			90-Day mortality	22
			1-Year mortality	53
			3-Year mortality	5
			5-Year mortality	59

Surgeon Age. To our knowledge, only one study to date, a nationwide Swedish cohort study of 139 surgeons, has evaluated surgeon age in relation to long-term survival after esophagectomy for cancer. Younger and older surgeons tended to have worse long-term outcomes than those with ages in-between, indicating a short “surgeon age peak performance” between 52 and 56 years, which remained after adjustment for established prognostic factors (i.e., tumor stage, patient age, comorbidity, and complications).^28^

Timing of Surgery. A population-based study from Sweden (1748 patients) indicated that surgery on a later weekday reduces long-term survival after surgery for esophageal cancer after adjustment for relevant confounders.^29^ The overall risk of 5-year mortality increased for each later weekday (HR, 1.13; 95% CI, 1.01–1.26) and was higher for early tumor stages (HR, 1.24; 95% CI, 1.09–1.41) and absent for advanced tumor stages (HR, 0.98; 95% CI, 0.92–1.05). Another study from Sweden using a different design and study period found similar results.^30^ However, no such association was identified in a Dutch register-based study (3840 patients).^31^ Patients who underwent esophagectomy during holiday periods did not seem to have any worse survival than those who had surgery during non-holiday periods according to two population-based and nationwide Swedish cohort studies.^32^^,^^33^

Summary of Surgeon-Related Factors. Surgeon volume seems to have a substantial influence on the chance of long-term survival after esophagectomy for esophageal cancer. Proficiency gain curves may be long before surgeons achieve stable long-term survival for their patients, possibly longer if the training period is less intensive and if the surgeon is older. Surgeon age and weekday of surgery also might be prognostic factors, although evidence is too limited for any strong conclusions to be drawn.

## Discussion

The existing literature has generally shown only small or no differences in long-term survival depending on variations in the surgical approach for esophageal cancer, whereas some direct surgeon-related factors may have a greater impact on the life expectancy for these patients.

Current evidence indicates a critical role for the skills of the individual esophageal surgeon, probably more than for the surgical approach when it comes to survival in esophageal cancer. From an esophageal cancer patient point of view, undergoing surgery by a highly qualified and experienced high-volume surgeon optimizes the chance of cure independently, it seems, of whichever surgical approach the surgeon prefers. These findings indicate a need to pay more attention to how the skills of the individual esophageal cancer surgeon are achieved and maintained. These challenges are not specific to esophageal cancer surgery but are more widespread with the introduction of advanced minimal access techniques, increasing the complexity and potential patient harm during the surgical learning curve.

Careful selection of the most suitable candidates seems justified. Appropriate and structured training programs at specialized and large centers for conducting these demanding procedures are warranted. Introduction of objective assessments with feedback of anonymous peers (e.g., evaluation of random video-recorded procedures with human reliability analysis) may help in assessing and maintaining the required surgical standards of the individual esophageal cancer surgeon.

To reduce patient harm during the period of proficiency gain for surgeons, well-structured training programs before independent practice of esophagectomy are urgently needed. Future surgical training programs may be based on competency-based assessments before initiation of independent practice to replace current training systems largely based on the number of procedures, years of training, and subjective trainers’ opinions. This type of competency-based training curriculum should be followed by a period of mentorship and continual technical proficiency assessment to support esophageal surgery specialists during the initial phases of their independent practice.

Centralization of esophagectomy for cancer to high-volume surgeons has clearly been shown to improve survival.^19^^,^^20^ An additional benefit of concentrating esophagectomy practice may include increased frequency of surgeon practice, thus reducing the period of proficiency gain.^27^ Importantly, centralization of esophageal cancer surgical services to fewer high-volume surgeons working at large centers also will facilitate research and development as well as continual assessments by experienced colleagues to optimize surgical performance of demanding procedures such as esophagectomy.

Several ongoing esophageal cancer trials are focused on optimizing neoadjuvant oncological therapy.^34^^,^^35^ This clearly is good, but the surgical and surgeon-related factors highlighted in this review are associated with variations in patient survival larger than those seen with the use of neoadjuvant chemoradiotherapy.^36^

The importance of controlling surgical quality in RCTs also has been demonstrated, with influence on mortality and locoregional recurrence.^37^ Therefore, future research needs to identify strategies that lead to improvement of surgical and surgeon-related factors inside clinical trials, and perhaps more importantly, in real clinical practice.

## Conclusions

Esophagectomy is the mainstay of curative treatment for most patients with a diagnosis of esophageal cancer. Generally, RCTs comparing various surgical approaches of esophagectomy have failed to identify any major differences in long-term prognoses. Recent large cohort studies have identified some direct surgeon-related factors that seem to strongly influence the long-term survival in esophageal cancer, including surgeon volume and the proficiency gain period, and possibly also surgeon age and timing of surgery. Future research is necessary to evaluate modification of these factors and to determine how this in turn may improve long-term survival in esophageal cancer both inside and outside a clinical trial setting.
